# Morphology, Thermal, Mechanical Properties and Rheological Behavior of Biodegradable Poly(butylene succinate)/poly(lactic acid) In-Situ Submicrofibrillar Composites

**DOI:** 10.3390/ma11122422

**Published:** 2018-11-30

**Authors:** Zhiwen Zhu, Hezhi He, Bin Xue, Zhiming Zhan, Guozhen Wang, Ming Chen

**Affiliations:** 1 National Engineering Research Center of Novel Equipment for Polymer Processing, South China University of Technology, Guangzhou 510640, China; mrobito@163.com (Z.Z.); xbinscut@163.com (B.X.); 201721002896@mail.scut.edu.cn (Z.Z.); gjoewang@foxmail.com (G.W.); chenm243@163.com (M.C.); 2 Key Laboratory of Polymer Processing Engineering, Ministry of Education, South China University of Technology, Guangzhou 510640, China

**Keywords:** biodegradable, poly(lactic acid) submicro-fibrils, storage modulus, crystallization

## Abstract

In this study, biodegradable poly(butylene succinate)/poly(lactic acid) (PBS/PLA) in-situ submicrofibrillar composites with various PLA content were successfully produced by a triple-screw extruder followed by a hot stretching−cold drawing−compression molding process. This study aimed to investigate the effects of dispersed PLA submicro-fibrils on the thermal, mechanical and rheological properties of PBS/PLA composites. Morphological observations demonstrated that the PLA phases are fibrillated to submicro-fibrils in the PBS/PLA composites, and all the PLA submicro-fibrils produced seem to have a uniform diameter of about 200nm. As rheological measurements revealed, at low frequencies, the storage modulus (*G’*) of PBS/PLA composites has been increased by more than four orders of magnitude with the inclusion of high concentrations (15 wt % and 20 wt %) of PLA submicro-fibrils, which indicates a significant improvement in the elastic responses of PBS melt. Dynamic Mechanical Analysis (DMA) results showed that the glass transition temperature (*T_g_*) of PBS phase slightly shifted to the higher temperature after the inclusion of PLA. DSC experiments proved that fiber morphology of PLA has obvious heterogeneous nucleation effect on the crystallization of PBS. The tensile properties of the PBS/PLA in-situ submicrofibrillar composites are also improved compared to neat PBS.

## 1. Introduction

Presently, the lack of petroleum resource and environmental pollution issues have become increasingly prominent. White pollution caused by non-degradable plastics has attracted a series of interests in the research of biodegradable polymer composites [1]. Due to the similar physical characteristics of poly(butylene succinate) (PBS) and low density polyethylene (LDPE) [2,3], biodegradable PBS is one of the best prospected materials to replace conventional nonbiodegradable petroleum-based LDPE material [4]. Nevertheless, the extensive use of PBS has been seriously restricted by its defects including low melt viscosity and insufficient stiffness [5].

Considering that PBS is a semicrystalline polymer, the crystalline architecture has a profound effect on its properties. Several approaches have been developed to improve PBS’s properties. Copolymerization with other dicarboxylic acids [6,7,8] or diols [4,9] is an effective strategy for amending the properties of PBS to extend its application [10]. The addition of different nano-fillers or fibres, such as layered silicate [11], graphene nanosheets (GNS) [12], NaY zeolite [13], jute fibre [14] and coir fiber [15], have been considered to tailor the crystallization behavior, mechanical and thermal properties of PBS with certain success. For instance, Hu et al. [12] investigated the influences of dispersing GNS on the property of PBS nanocomposites, and reported that the tensile strength of PBS nanocomposites increased by about 21% with the inclusionof 2.0 wt % of GNS. However, the effectiveness of incorporating nano-fillers or fibres on the properties of composites depends largely on the dispersion of these fillers in composites [16]. Although nanocomposites have been extensively studied, the uniform dispersion of fillers still faces many challenges [17]. Poly(lactic acid) (PLA) is a biobased and biodegradable aliphaticthermoplastic polyester which is synthesized from reproducible resources via lactic acid zymplysis [18], and virgin PLA has a strong but brittle nature. Owing to complementary property of PLA and PBS: the toughness of PBS and the stiffness of PLA, the PLA/PBS blends have been attracting more and more interest from industry and academia. For instance, Fortunati et al. [19] investigated PLA/PBS composites with different plasticizers and reported that the PLA/PBS-based film plasticized by isosorbide is a environmental friendly material for packaging application. However, as far as the authors known, a great majority of these studies are aimed to tailor the properties of PLA.

The morphology of the dispersed phase in immiscible polymer systems has a profound effect on the properties of polymer blends [20]. Owing to the fibrillar morphology of dispersed phase shows enormous potential to enhance the property of several polymer blends [21,22,23,24,25,26,27,28]. Microfibrillar composites (MFCs) featured by the fibrillar morphology of dispersed phase recently have received considerable interest. Currently, the fabrication process of MFCs is defined as follows: (a) Melt extrusion: blend of matrix and dispersed component is conducted using conventional melt extrusion equipment, the melting point (*T_m_*) of the dispersed polymer (*T*_1_) is at least 40 °C higher than that of the matrix (*T*_2_) [17]. (b) Fibrillization: transform the dispersed phase from droplet morphology into fibrillar morphology [21], either in molten (hot-stretching) or solid (cold-drawing) states. (c) Isotropization: then the blends are processed at *T*_2_ < *T* < *T*_1_ to prepare MFCs while the fibrillar morphology of dispersed phases is preserved.

Recently, PBS/PLA composites produced by slit die extrusion−stretching−woven compression molding have been shown [29]. It is reported that the PLA submicro-fibrils tend to form an interwoven network through a weaving and compression molding process, which provides better gas barrier property and enhanced strength and ductility. However, as far as the authors known, there is no intensive studies that have been investigated the impact of PLA submicro-fibrils on the thermal and rheological properties of PBS/PLA in-situ submicrofibrillar composites without the influence of woven hot compaction. In this work, biodegradable PBS/PLA in-situ submicrofibrillar composites have been successfully produced by a triple-screw extruder following a hot stretching−cold drawing−compression molding process. The aim of the present work is to study the influences of PLA submicro-fibrils on the thermal, mechanical and heological properties of PBS/PLA composites.

## 2. Materials and Methods

### 2.1. Materials

The matrix polymer adopted in this study, PBS with a number-average-molecular-weight of nearly 6.0 × 10^4^ g/mol and a melt-flow-index (MFI) of 1.5 g/10 min (190 °C/2.16 kg), is purchased from Showa Polymer Co. (Tokyo, Japan) Bionolle 1001MD. Commercially available PLA (trade name 4032D) was purchased from Nature Works Co. (Blair, NE, USA). This product has a weight-average-molecular-weight of 2.23 × 10^5^ g/mol, a MFI of 7 g/10 min (210 °C/2.16 kg). The melting temperatures (*T_m_*) of PBS and PLA were tested by Differential Scanning Calorimetry experiments. The *T_m_* of PBS and PLA are 114 °C and 168 °C, respectively.

### 2.2. Preparation of PBS/PLA Composites

**Figure 1:**
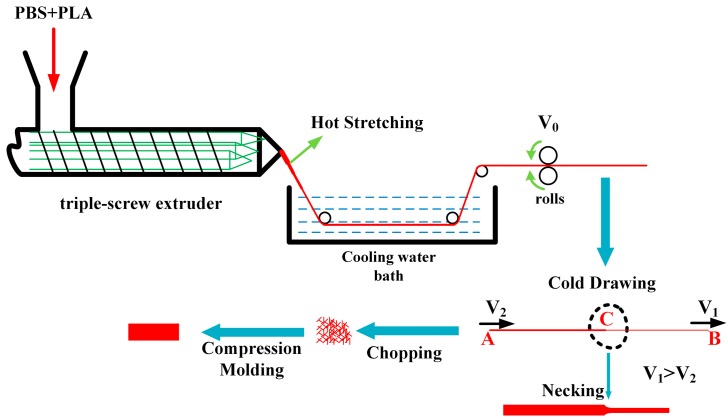
Scheme of the process for PBS/PLA in-situ submicrofibrillar composites preparation.

The PBS/PLA composites were fabricated following a hot stretching−cold drawing−compression molding process. Prior to being processed, PBS and PLA were dried at 100 °C for 12 h in a vacuum oven. The PBS/PLA blends with mass ratios of 97/3, 95/5, 90/10, 85/15 and 80/20 were fabricated through a triple-screw extruder (manufactured by Guangzhou POTOP Co. Ltd., Guangzhou, China) with a screw diameter of 25 mm and a aspect ratio of 40. The screw speed was set at 100 rpm and the temperature profile was 90 °C, 130 °C, 165 °C, 165 °C, 165 °C, 165 °C, 165 °C, 165 ℃ and 165 °C. The scheme for the preparation of PBS/PLA submicrofibrillar composites is depicted in Figure 1. The extrudates were hot-stretched to a diameter of about 0.37 mm at the round die exit (diameter is 2.5 mm) and were promptly quenched in cooled water for solidification. Subsequently, the hot-stretched extrudates were cold-drawn (the velocity of B Point (*V1*) is greater than that of A Point (*V2*): *V1* > *V2*) at room temperature (RT 20 °C~30 °C). As shown in Figure 1, necking was observed during cold drawing. Eventually, after completion of the necking process, the extrudates are too stretched to undergo stretching. It is of great interest that the diameter of the cold-drawn extrudate is reduced to nearly 0.17 mm for PBS/PLA blends with different PLA content. The samples were then chopped into small rods with length of about 3 mm and compressed into a flat sheet using a laboratory hydraulic press (KS100HR, Dongguan City Kesheng Machinery Co. Ltd., Dongguan, China) at 130 °C under a pressure of 10 MPa for 10 min followed by cooling down to the RT.

Neat PBS was also treated using the same conditions as that for PBS/PLA composites to maintain a consistent thermal/processing history. The obtained final composites were named as the cold-drawn PBS/PLA. For comparison, the as-extruded PBS/PLA (PBS/PLA blend compression-molded right after the extruder) were also fabricated.

### 2.3. Scanning Electron Microscopy (SEM)

PBS/PLA composites were placed in liquid nitrogen for 1 h and then cryo-fractured before observation. The cryo-fractured surfaces were coated with a thin gold layer. Morphology of PBS/PLA composites was observed using a Quanta FEG 250 SEM (Thermo Fisher Scientific, Hillsboro, OR, USA). The diameters of PLA phases were determined from SEM micrographs by using Image-Pro Plus 6.0 software (Version 6.0.0.260). At least 200 PLA domains for each sample were characterized for statistical analysis.

### 2.4. Rheological Characterization Measurements

The linear viscoelastic responses of PBS and PBS/PLA composites were studied using a controlled-strain rheometer (Physica MCR 302, Anton Paar, Graz, Austria). The experiments were conducted at 130 °C, a temperature between the *T_m_* of PBS (*T_m_* = 114 °C) and PLA (*T_m_* = 168 °C), to melt the PBS phase but keep the original phase morphology of PLA during test. Frequency sweeping between 0.0628 and 628 rad/s were conducted at a controlled strain of 1%, which is within the linear viscoelastic range.

### 2.5. Dynamic Mechanical Analysis (DMA)

DMA was performed on a TA Q800 (New Castle, DE, USA) instrument under a tensile mode. The frequency of 2 Hz and oscillating strain. of 15 mm was used. The scanning rate was 3 °C/min in the range of −80 °C to +50 °C. The glass transition temperature (*T_g_*) was determined by the summit of loss tangent (tanδ) peaks.

### 2.6. Differential Scanning Calorimetry (DSC) Measurements

The melting and crystallization behaviors of PBS and PBS/PLA composites were studied using DSC (DSC204F3, Netzch, Germany). DSC experiments were conducted under nitrogen atmosphere to avoid oxidation. The specimens were firstly heated to 135 °C and held at the targeting temperature for 3 min to remove thermal and stress histories, and then cooled down to 30 °C. *T_m_* of PBS phase was determined during the specimens were again heated to 135 °C. The ramp rates were 5 °C/min for both the heating and cooling sessions. Crystallinity (*X_c_*) of PBS in composites was determined by using the following equation:

(1) $$X_{c}=(\frac{\Delta H_{m}-\Delta H_{c}}{\Delta H_{o}\times \omega })\times 100\%$$

where *ΔH_m_* and *ΔH_c_* are the melting enthalpy and cold crystallization enthalpy of PBS phases, respectively. And *ΔH_o_* is the melting enthalpy for a 100% crystalline of PBS, ω represents the PLA weight fraction in the blends. For PBS, *ΔH_o_* is 120 J/g [29]. Five specimens were tested for every PBS/PLA composite and the average values and errors were calculated.

### 2.7. Wide-Angle X-ray Diffraction (WAXD)

WAXD experiments of neat PBS and PBS/PLA composites were conducted on a Xenocs Xeuss 2.0 system with an Excillum MetalJet-D2 X-ray source and a detector (Pilatus 3R 1M, Dectris, Baden, Switzerland). The sample-to-detector distance of 222.069 mm and a wavelength of 0.134144 nm were used.

### 2.8. Tensile Tests

According to the ISO 527 standard test method, the tensile properties of the compression-molded composites were tested at RT by using an Instron Universal Testing Machine (model 5566, Norwood, MA, USA). The crosshead speed was 5 mm/min. At least five dumbbell-shaped samples were tested and mean values were calculated.

## 3. Results and Discussions

### 3.1. Phase Morphology of PBS/PLA Composites

**Figure 2:**
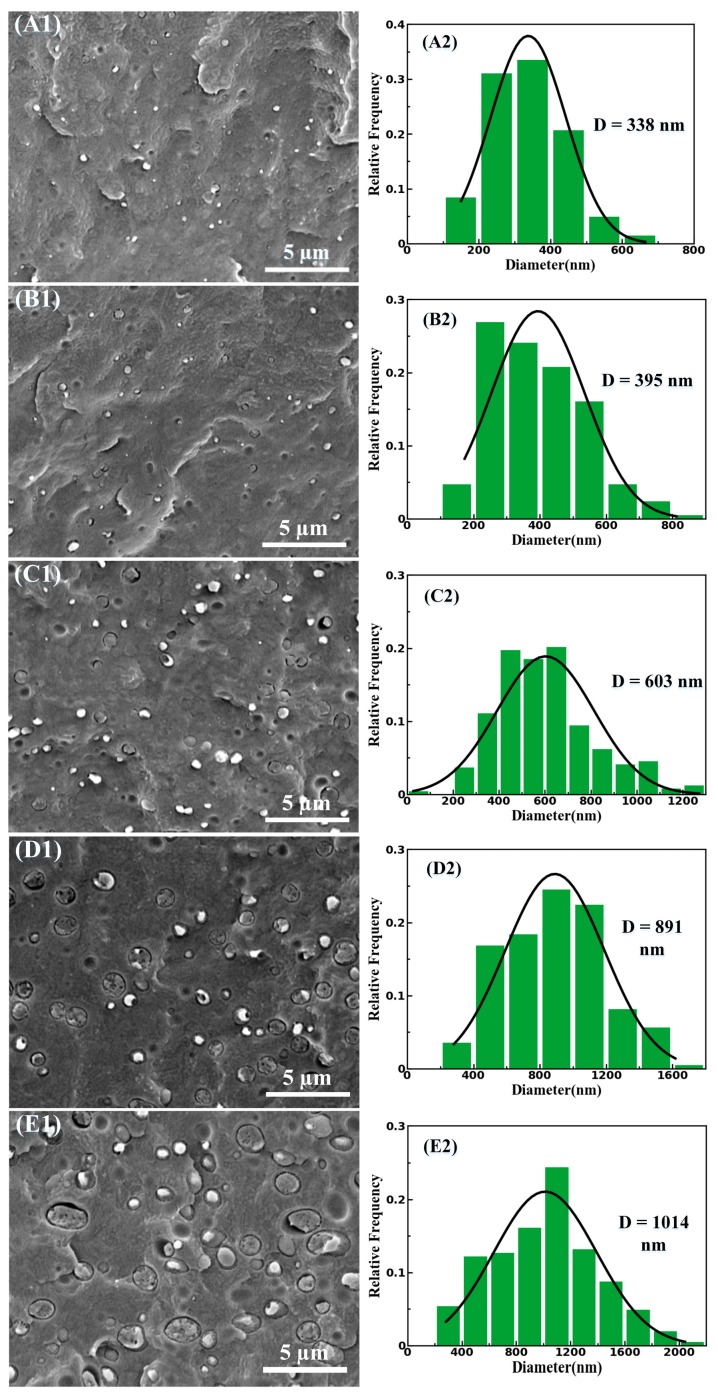
SEM micrographs of the cryo-fractured surfaces of the as-extruded PBS/PLA composites with 3 wt % (**A1**), 5 wt % (**B1**), 10 wt % (**C1**), 15 wt % (**D1**), and 20 wt % (**E1**) of PLA. The statistical analyses with respect to the distribution of the diameters of PLA domains as shown in (**A2**), (**B2**), (**C2**), (**D2**) and (**E2**), respectively. D refers to the average diameter of PLA domains.

As is known to all, the morphology of dispersed phase of polymer blend is profoundly affected by the blend composition, nature of flow field, processing condition, rheological and interfacial properties, viscosity ratio, and other factors [30]. Figure 2 shows the SEM micrographs of cryo-fractured surfaces for the as-extruded PBS/PLA with various PLA concentrations, and the corresponding diameter distribution of the PLA domains. It is observed that the PLA phases are more uniformly dispersed in the PBS matrix and a representative two-phase morphology comprising discontinuous domains of the lesser phase dispersed in the continuous major phase is evidently presented for the PBS/PLA composites (Figure 2A1–E1), this is attributed to the thermodynamic incompatibility between PBS and PLA which is consistent with the observation of other investigators [3,31]. The average diameter of the PLA droplets ranged from nearly 338 nm, under the circumstance of the PBS/PLA (97/3) composites, to around 1014 nm, under the circumstance of the PBS/PLA (80/20) composites. As the final diameter of dispersed particles is the result of the equilibrium between their breakup and coalescence [32], the average diameter of the PLA droplets increases dramatically at high concentrations, which is ascribed to the enhanced agglomeration possibility of PLA droplets. Compared with the poly (phenylene sulfide)/isotactic polypropylene (PPS/iPP) [32], polypropylene/polyethylene terephthalate (PP/PET) [22,28,30] and other immiscible systems, in which the dimension of dispersed particles is in the range from several to hundreds of micrometer, the small dimensions of dispersed PLA particles can be the result of the intensive shearing flow field of the triple-screw extruder and the decrease of the probability of coalescence in the PLA domains owing to the low processing temperature(165 ℃).

**Figure 3:**
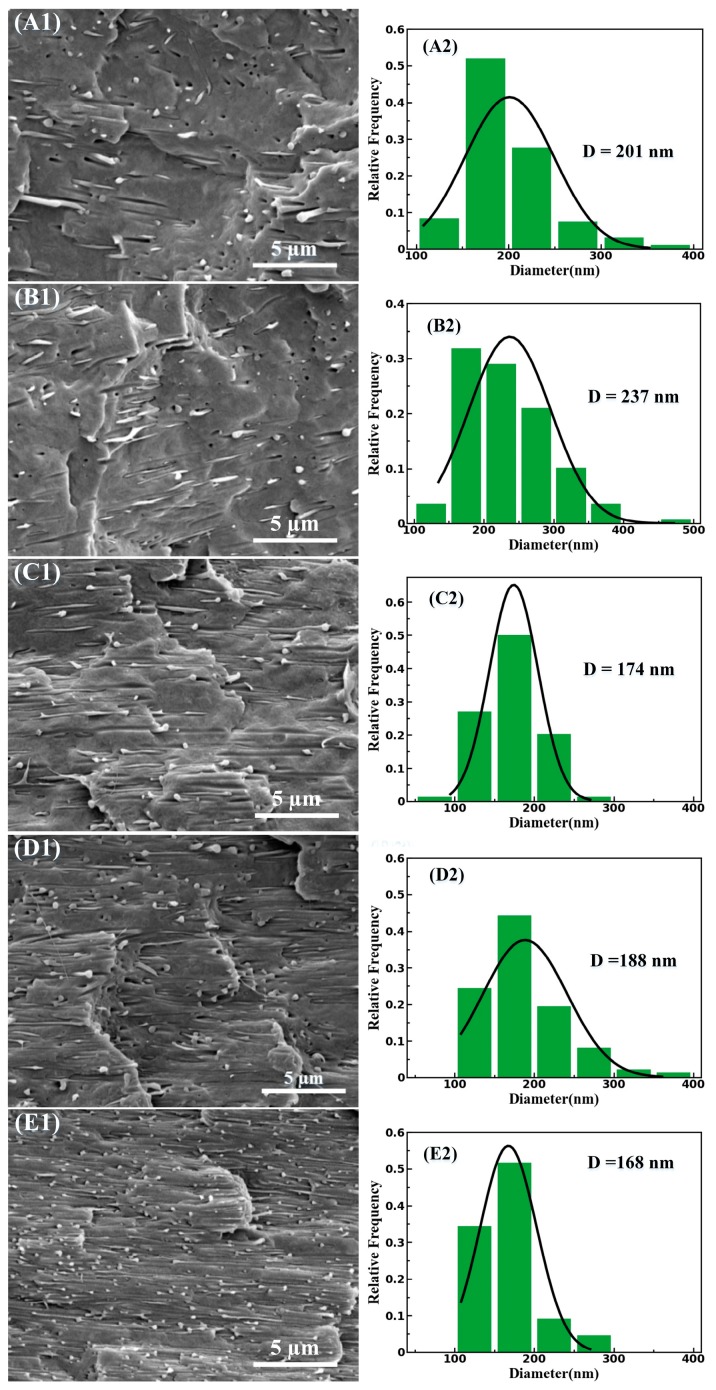
SEM micrographs of the cryo-fractured surfaces of the cold-drawn PBS/PLA composites with 3 wt % (**A1**), 5 wt % (**B1**), 10 wt % (**C1**), 15 wt % (**D1**), and 20 wt % (**E1**) of PLA. The statistical analyses with respect to the distribution of the diameters of PLA domains as shown in (**A2**), (**B2**), (**C2**), (**D2**) and (**E2**), respectively. D refers to the average diameter of PLA domains.

As shown in Figure 3, the PBS phase is converted into an isotropic matrix and the PLA phase has preserved its elongational orientation in all the cold-drawn PBS/PLA composites after compression molding process. The great majority of the PLA phase in the cold-drawn PBS/PLA (97/3) composites in the form of fibril were observed to be well-distributed in PBS matrix, as shown in Figure 3A1. In addition, as shown in Figure 3B1–E1, the diameter of the dispersed PLA fibrils decreases and the length of the dispersed PLA fibrils increases after cold drawing. The statistical analyses with respect to the diameter distribution of PLA fibrils in the cold-drawn PBS/PLA composites as indicated in Figure 3A2–E2. The average diameters of PLA fibrils ranged from about 201 nm, under the circumstance of the cold-drawn PBS/PLA (97/3) composites, to nearly 168 nm, under the circumstance of the cold-drawn PBS/PLA (80/20) composites. It is remarkable that the PLA fibrils show nano-sized diameter and have a similar average diameter of about 200 nm after cold drawing. The similar average diameter may be ascribed to the hindrance of the coalescence of deformed PLA domains, due to the low temperature during the cold-drawing process. It is of great importance that the degree of dispersion and distribution of PLA fibrils do not decrease with the increase of PLA content because of the uniform dispersion of PLA domains in the PBS matrix for the as-extruded PBS/PLA composites. A greater degree of fibrillation can be observed after the cold-drawing process, which can be put down to the following two causes: for one thing, an elongational flow field generated by the cold drawing process should be responsible for further increasing of the aspect ratio of the dispersed PLA domains. For another, as the cold-drawing process is carried out at RT of about 27 °C, which is lower than the *T_g_* of PLA (62.6 °C), the fibrillar structure of the dispersed PLA phase can be preserved without recoiling and the coalescence of deformed PLA domains is hindered. Consequently, the aspect ratio of PLA fibril, which is a crucial parameter for the characterization of fibril, has been greatly improved after cold drawing process. Unfortunately, is an arduous task to accurately calculate the aspect ratio of the PLA fibrils, as it is impossible to distinguish or separate the two phases in PBS/PLA composites.

### 3.2. Rheological Behaviors

**Figure 4:**
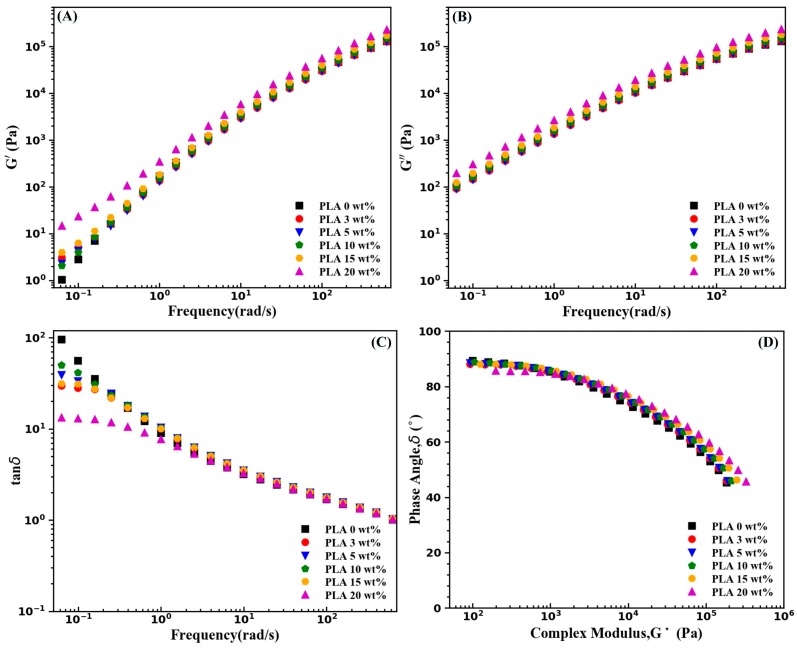
Rheological behavior of the as-extruded PBS/PLA composites at 130 °C; (**A**) Frequency dependence of G’(ω); (**B**) Frequency dependence of G’’(ω); (**C**) Frequency dependence of tanδ; (**D**) Van Gurp plots.

It is well known that rheological behaviors of polymer composites can be used to investigate their microstructures [33]. Figure 4 shows shear responses of neat PBS and the as-extruded PBS/PLA composites. As shown in Figure 4A, at low frequencies, increasing the concentration of PLA brings about a gradual increase in storage modulus (*G’*) of PBS, which can be ascribed to the effect of solid PLA phases on the stress relaxation behavior of the PBS melt. However, the PLA phases of up to 15 wt % only caused a slight increase in the *G’* of PBS and only the high content of PLA (20 wt %) led to a higher increase in the *G’* of PBS. The frequency dependence of viscous modulus (*G’’*) is depicted in Figure 4B, and the discrepancy of *G’’* of the as-extruded PBS/PLA composites is very small over the studied frequency range. The semilogarithmic plots of loss tangent (tanδ) with respect to frequency and phase angle with respect to complex modulus (G*) (Van Gurp polts) were depicted in Figure 4C,D, respectively. At low frequencies, the tanδ values decrease with the inclusion of PLA, which can be interpreted as a lower viscous/elastic ratio. Nevertheless, as shown in Figure 4D, the phase angle of nearly 90° is observed for the neat PBS and all the as-extruded PBS/PLA composites, at low complex moduli, which illustrates the dominance of the viscous behavior.

**Figure 5:**
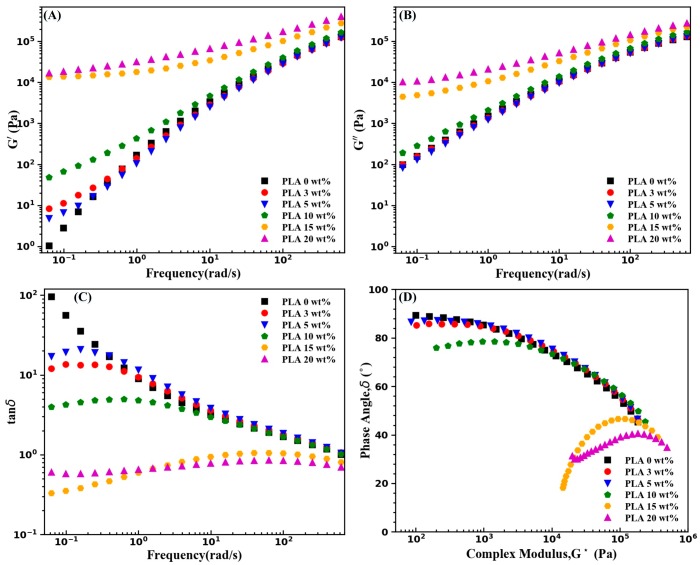
Rheological behavior of the cold-drawn PBS/PLA composites at 130 ℃; (**A**) Frequency dependence of G’(ω); (**B**) Frequency dependence of G’’(ω); (**C**) Frequency dependence of tanδ; (**D**) Van Gurp plots.

Shear responses of the cold-drawn PBS/PLA composites at 130 °C are presented in Figure 5. Figure 5A shows frequency-dependence of the *G’* in the low frequency region remarkably decreases with increasing PLA concentration. The increase in the *G’* of samples with fibril morphology of PLA is more striking than that of samples with PLA droplets, which illustrates that compared to the PLA droplets, the PLA submicro-fibrils have higher effect on improving the elastic behavior of PBS. Specially, inclusion of high concentrations (15 wt % and 25 wt %) of PLA submicro-fibrils increased the *G’* by over four orders of magnitude, indicating a prominent increase in the elastic responses of PBS melt. This can be explained by the restrictions in the motion of PBS chains, which are brought about by the physical constraint effect of the solid-state PLA submicro-fibrils. However, in respect to samples of the cold-drawn PBS/PLA (97/3) composites and the cold-drawn PBS/PLA composites (95/5), the *G’* has an outstanding dependence on frequency in the low frequency range, which is an evidence of the predominance of viscous behavior in PBS melt at low frequencies. Furthermore, as shown in Figure 5B, the effect of PLA submicro-fibrils on the *G’’* is less notable than that on the *G’*. The plot of tanδ with respect to frequency is presented in Figure 5C. It is worthy of mentioning that the tanδ of the cold-drawn PBS/PLA composites becomes independent of frequency at low frequencies. For samples with PLA submicro-fibril contents of 10 wt % and less, the tanδ monotonically decreases with an increase in frequency, which demonstrates the samples are still a liquid-like system. It is seen that the tanδ values of samples with high concentrations (15 wt % and 20 wt %) of PLA submicro-fibrils drop to less than 1, indicating that the viscoelastic behavior of samples is varying from liquid-like to gel-like state. The Van Gurp plot, as illustrated in Figure 5D, also indicates that the phase angle of samples with high concentrations (i.e., 15 wt % and 20 wt %) of PLA submicro-fibrils is considerably lower than that of neat PBS at low complex moduli.

In the light of the Winter and Chambon [34], tanδ is independent of frequency at the gel point where physical or chemical gelation occurs [21]. Therefore, a PLA submicro-fibril content between 10 and 15 wt % should be the gel point of the PBS/PLA composites. The high gel point concentration of the PBS/PLA composites can be attributed to the following two factors: for one thing, as shown in Figure 3A2–E2, the PLA submicro-fibrils have a high degree of orientation and no reticular formation after the compression molding step. For another, the aspect ratio of fibrils has an enormous impact on the relaxation process of the polymer chains. Although it is still arduous to calculate the aspect ratio of the PLA fibrils accurately, it is shown in Figure 3A2–E2 that the PLA submicro-fibrils seemingly have no great aspect ratios.

### 3.3. Thermal Properties

**Figure 6:**
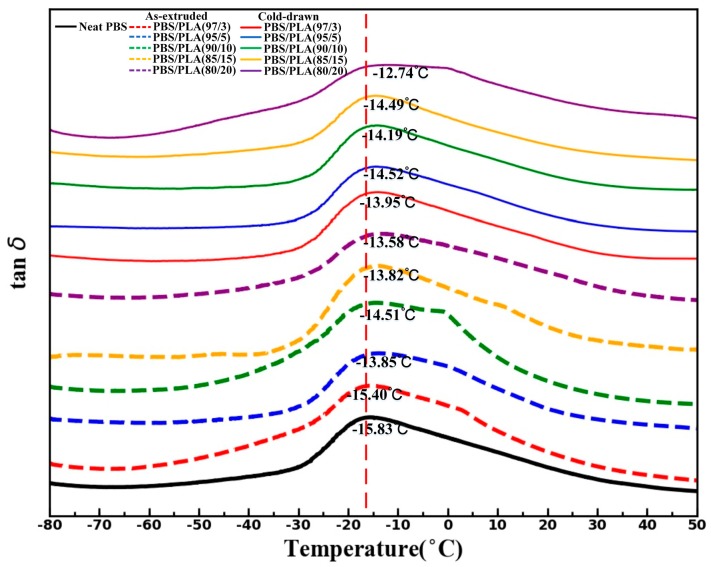
DMA curves of the neat PBS, the as-extruded PBS/PLA composites and the cold-drawn PBS/PLA composites.

The *T_g_* of PBS can be varied with the addition of dispersed phases [35]. DMA experiments were conducted to observe the change of *T_g_* of PBS phase in PBS/PLA composites. As illustrated in Figure 6, an apparent peak of tanδ is exhibited about −15.83 ℃ which is attributed to glass transition of neat PBS. With the inclusion of PLA, a small positive shift in the tanδ peak can be observed in PBS/PLA composites, which indicating that the PLA can slightly hinder the mobility of PBS segments. In addition, the morphology of PLA and PLA content have little influence on the *T_g_* of PBS component in PBS/PLA composites. The small effect of PLA on the *T_g_* of PBS phase may be ascribed to the incompatibility between PBS and PLA, as illustrated in the SEM micrographs (Figure 2 and Figure 3), which results in the poor interaction between the two phases.

**Figure 7:**
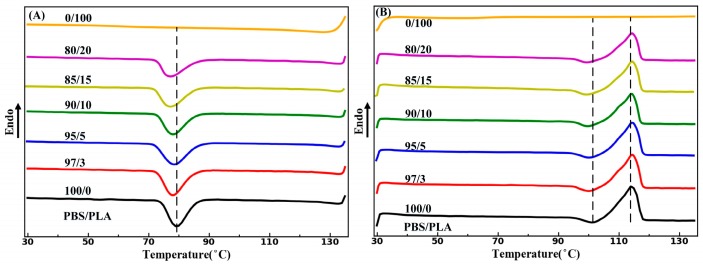
DSC curves of the neat PBS, PLA and the as-extruded PBS/PLA composites with various PLA concentrations: (**A**) first cooling curves and (**B**) second heating curves.

DSC was employed to investigate the influence of PLA morphology on the crystallization property of PBS. DSC curves for neat PBS, neat PLA and the as-extruded PBS/PLA composites were plotted in Figure 7. Crystallization temperature (*T_c_*), cold crystallization temperature (*T_cc_*), cold crystallization enthalpy (*ΔH_cc_*), *T_m_*, melting enthalpy (*ΔH_m_*) and the degree of crystallinity (*X_c_*) were tabulated in Table 1. It shows in Figure 7, that no obvious endothermic or exothermic events could be observed on the cooling process and heating processes of neat PLA. Consequently, enthalpy change of PLA in the PBS/PLA composites has almost no effect on the DSC thermograms of the PBS phase. As shown in Figure 7A, PBS phase in all the as-extruded PBS/PLA composites shows almost the same *T_c_*, which is slightly lower than that of neat PBS. It reveals that the PLA phase of the as-extruded PBS/PLA composites has almost no heterogeneous nucleation effect on the crystallization of PBS. In addition, it is seen in Table 1 that both the neat PBS and the PBS phase in the as-extruded PBS/PLA composites show nearly the same *T_m_*. Moreover, the *X_c_* of PBS phase has little dependence on the PLA droplet content.

**Figure 8:**
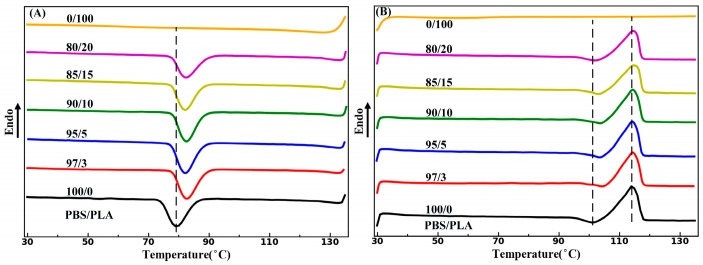
DSC curves of the neat PBS, PLA and the cold-drawn PBS/PLA composites with various PLA concentrations: (**A**) first cooling curves and (**B**) second heating curves.

Contrary to the DSC curves of the as-extruded PBS/PLA composites as mentioned above, an obvious increase in *T_c_* is observed in the DSC curves of all the cold-drawn PBS/PLA composites in Figure 8A. As shown in Table 2, the *T_c_* of the cold-drawn PBS/PLA (97/3) composites increases by nearly 3 °C compared with that of neat PBS, which means that fiber morphology of PLA has obvious heterogeneous nucleation influence on the crystallization property of PBS. Therefore, the heterogeneous nucleation influence of PLA on the crystallization behavior of PBS is related to the morphology of PLA. The *T_m_* of PBS phase in the cold-drawn PBS/PLA composites has a slightly increase with the increase of PLA content. Furthermore, the *X_c_* of PBS of the cold-drawn PBS/PLA composites has also limited dependence on the PLA fibril content, which is almost the same as PBS of the as-extruded PBS/PLA composites. It is noteworthy that PLA with fiber morphology has obvious heterogeneous nucleation influence on crystallization behavior of PBS, while PLA with droplet morphology has no such effect, which may be attributed to a more heterogeneous crystal nucleation sites of fiber morphology [21,22].

**Figure 9:**
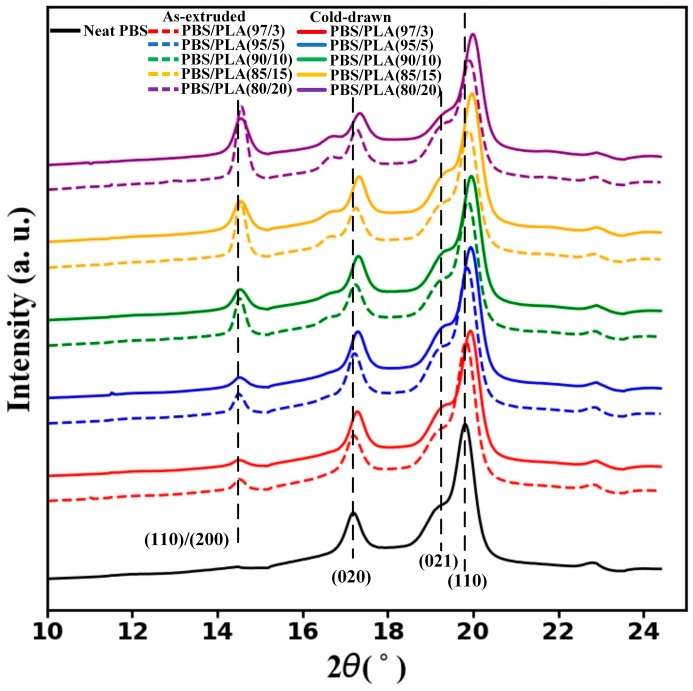
1D-WAXD diffraction profiles of neat PBS and PBS/PLA composites.

To further investigate the effect of PLA morphology on PBS crystallization, WAXD measurements were conducted. The 1D-WAXD diffraction profiles obtained by azimuthally integration of 2D-WAXD patterns of neat PBS and PBS/PLA composites are illustrated in Figure 9. The reflections of (020), (021) and (110) for the α-crystals of PBS are respectively observed, which is consistent with the observation of Guo’s research [36]. And The reflection of (110)/(200) refers to the PLA α’-form [37]. All the curves of the PBS/PLA composites have the same reflections for the α-crystals of PBS as the neat PBS. And there is no difference in the type of crystalline phases of PBS between the as-extruded PBS/PLA composites and the cold-drawn PBS/PLA composites. All of this indicates neither the fiber morphology of PLA nor the droplet morphology of PLA have influence on the type of crystalline phases of PBS.

### 3.4. Mechanical Properties

**Figure 10:**
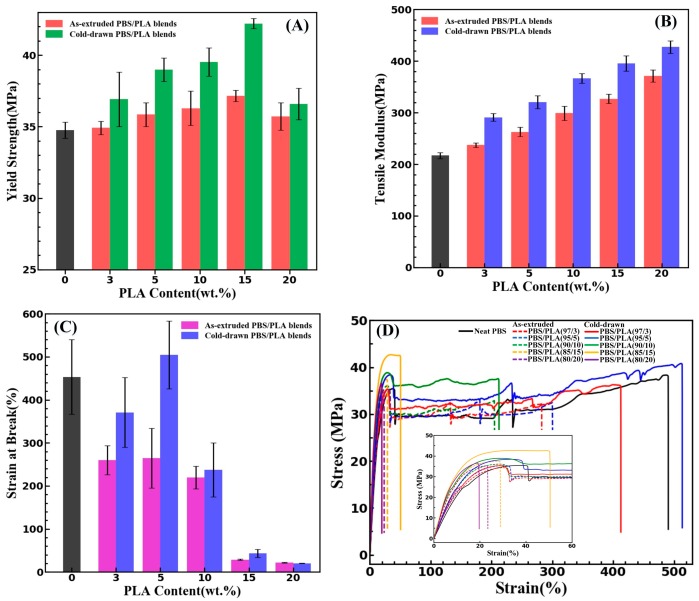
Yield strength (**A**), tensile modulus (**B**), strain at break (**C**), and stress-strain curves (**D**) of the as-extruded PBS/PLA composites and the cold-drawn PBS/PLA composites with various PLA concentrations.

The effect of PLA submicro-fibrils on the tensile properties of the PBS/PLA composites has been studied. As shown in Figure 10A, it reveals that the yield strength of the as-extruded PBS/PLA composites gradually increases to its peak at concentration of 15 wt % for PLA. In comparison with the as-extruded PBS/PLA composites, the cold-drawn PBS/PLA composites have a substantially increase in the yield strength. When the concentration of PLA increases to 15 wt %, the yield strength of the cold-drawn PBS/PLA composites is dramatically enhanced to 42.22 MPa, which is higher than that of neat PBS by 21.5%. Under the condition of 20 wt % PLA content, the yield strength of the cold-drawn PBS/PLA composites decreases seriously, however, is still higher than that of neat PBS.

As illustrated in Figure 10B, an approximately linear relationship is obtained between the tensile modulus of the cold-drawn PBS/PLA composites and the PLA concentration. All the PBS/PLA composites showed higher modulus than neat PBS. The increasing tendency of tensile modulus of the cold-drawn PBS/PLA composites with PLA content is the same as that of the as-extruded PBS/PLA composites. It is found that the cold-drawn PBS/PLA composites exhibit higher tensile modulus than the as-extruded PBS/PLA composites. The yield strength and tensile modulus of PLA increased with the morphology of PLA phase changing from droplets to submicro-fibrils. For instance, the yield strength and tensile modulus of the cold-drawn PBS/PLA blends containing 15 wt % PLA are 13.62% and 20.94% higher than those of the as-extruded PBS/PLA blends. It reveals that the reinforcing effect of the fibrillar morphology of PLA on the tensile properties of PBS is better than that of the droplet morphology. As shown in Figure 10C, it is of great interest that the strain at break of the cold-drawn PBS/PLA (95/5) composites is higher than that of neat PBS. When the content of PLA is less than 15 wt %, compared with the as-extruded PBS/PLA composites, the strain at break of the cold-drawn PBS/PLA composites is greatly improved. With the increase of PLA concentration from 10 wt % to 15 wt %, the strain at break of all the PBS/PLA composites has a sharp drop. The significant decrease of strain at break is largely on account of the destruction of the structural integrity of PBS by lots of PLA phases and the intrinsic brittleness of PLA. The stress-strain curves of neat PBS and PBS/PLA composites were depicted in Figure 10D. Neat PBS shows a typical ductile mode of failure. The PBS/PLA composites with the content of PLA are lower than 15 wt % fractured in ductile mode and the cold-drawn PBS/PLA composites have better tensile properties. However, when the PLA content increased to 15 wt % and 20 wt %, the PBS/PLA composites failed in a brittle manner.

## 4. Conclusions

The effects of in situ generated PLA submicro-fibrils on the rheological, thermal and mechanical properties of PBS/PLA composites were studied in this work. Morphological observation of the cold-drawn PBS/PLA composites with various PLA content revealed that the PLA fibrils show submicro-sized diameter and the average diameters of all the PLA fibrils were close to 200 nm. A investigation of the linear viscoelastic responses of PBS/PLA composites showed that G’(ω) of samples with fiber morphology of PLA was substantially higher, especially at high concentrations, than that of samples with PLA droplets, which can be attributed to the more restrained polymer chain motion of PLA submicro-fibrils. However, using Winter Chambon criteria, a high gel point concentration of 10–15 wt % can be observed, which can be mainly ascribed to high orientation of PLA submicro-fibrils, low length of PLA submicro-fibrils and the semi-rigid chains of PLA. DMA results revealed that the *T_g_* of PBS phase shifted to the slightly higher temperature after the inclusion of PLA, however, the morphology of PLA and PLA content have little impact on the *T_g_* of PBS component in PBS/PLA composites. DSC studies proved that PLA phases with droplet morphology have almost no heterogeneous nucleation effect on the crystallization of PBS. Nevertheless, fiber morphology of PLA has obvious heterogeneous nucleation effect on the crystallization of PBS. The study concludes that although the effect of PLA on the crystallization of PBS can be changed by the changes in the morphology of PLA, in general, PLA has no profound effect on the crystallization of PBS. In addition, neither the fiber morphology of PLA nor the droplet morphology of PLA have influence on the type of crystalline phases of PBS according to the investigation of WAXD experiments. Furthermore, compared with PLA droplet, PLA submicro-fibrils have more profound effect on improving the yield strength and tensile modulus of PBS.

## Figures and Tables

**Table 1:** DSC parameters of PBS phase in neat PBS, PLA and the as-extruded PBS/PLA composites.

Samples	*TC* (°C)	*TCC* (°C)	*Tm* (°C)	*ΔHCC* (J/g)	*ΔHm* (J/g)	*XC* (%)
PBS	79.5 ± 0.3	101.2 ± 0.1	114.2 ± 0.1	6.04 ± 0.36	66.84 ± 0.44	50.7 ± 0.6
PBS/PLA (97/3)	78.0 ± 0.5	100.1 ± 0.2	114.4 ± 0.1	7.07 ± 0.51	66.80 ± 1.85	51.3 ± 1.1
PBS/PLA (95/5)	78.3 ± 0.4	99.9 ± 0.2	114.4 ± 0.1	6.61 ± 0.55	63.91 ± 1.21	50.3 ± 0.6
PBS/PLA (90/10)	78.1 ± 0.3	99.8 ± 0.2	144.3 ± 0.2	6.49 ± 0.55	59.20 ± 0.79	48.8 ± 0.7
PBS/PLA (85/15)	77.2 ± 0.4	99.4 ± 0.2	114.3 ± 0.1	6.72 ± 0.30	56.87 ± 0.75	49.2 ± 1.0
PBS/PLA (80/20)	77.0 ± 0.9	99.4 ± 0.5	114.2 ± 0.1	5.69 ± 0.67	52.47 ± 0.77	48.7 ± 0.9

**Table 2:** DSC parameters of PBS phase in neat PBS, PLA and the cold-drawn PBS/PLA composites.

Samples	*TC* (°C)	*TCC* (°C)	*Tm* (°C)	*ΔHCC* (J/g)	*ΔHm* (J/g)	*XC* (%)
PBS	79.5 ± 0.3	101.2 ± 0.1	114.2 ± 0.1	6.04 ± 0.36	66.84 ± 0.44	50.7 ± 0.6
PBS/PLA (97/3)	82.7 ± 0.1	103.9 ± 0.3	114.3 ± 0.1	2.95 ± 0.29	63.47 ± 1.17	52.0 ± 0.8
PBS/PLA (95/5)	82.3 ± 0.1	103.5 ± 0.3	114.2 ± 0.1	4.38 ± 0.66	63.32 ± 1.08	51.7 ± 1.2
PBS/PLA (90/10)	82.6 ± 0.1	103.5 ± 0.4	144.3 ± 0.2	4.10 ± 0.44	59.09 ± 1.30	50.9 ± 1.1
PBS/PLA (85/15)	82.1 ± 0.3	102.8 ± 0.2	114.8 ± 0.4	4.28 ± 0.11	54.91 ± 1.39	49.6 ± 1.4
PBS/PLA (80/20)	82.4 ± 0.1	102.0 ± 0.1	114.8 ± 0.3	4.16 ± 0.24	52.47 ± 0.90	50.3 ± 0.8
